# Ureteric stenting after uncomplicated ureteroscopy: a systematic review of surgeons’ motivations and patient experiences

**DOI:** 10.1111/bju.70063

**Published:** 2025-11-20

**Authors:** Israa Hussein, Simona Ippoliti, Alexander BCD Ng, Ranil Johann Boaz, Stefanie Croghan, Cameron Alexander, Arjun Nathan, Nikita Bhatt, Kevin Gerard Byrnes, Veeru Kasivisvanathan

**Affiliations:** ^1^ Western General Hospital Edinburgh UK; ^2^ Hull University Teaching Hospitals NHS Trust, Urology Hull UK; ^3^ Division of Surgery & Interventional Science University College London London UK; ^4^ Division of Surgery and Interventional Science, Centre for Urology Imaging, Prostate, AI and Surgical Studies (COMPASS) Research Group University College London London UK; ^5^ University College London London UK; ^6^ The Royal London Hospital London UK; ^7^ Norfolk and Norwich University Hospital NHS Trust, Urology Norwich UK; ^8^ Department of Urology Freeman Hospital Newcastle Upon Tyne UK; ^9^ Department of Urology Colchester General Hospital Colchester UK; ^10^ Cambridge University Hospitals NHS Foundation Trust, Urology Cambridge UK

**Keywords:** urolithiasis, ureteroscopy, renal stone, ureteric stones, stenting

**Abbreviations**
EAU: European Association of Urology
NICE: National Institute for Health and Care Excellence
RCT: randomised controlled trial
URS: ureteroscopy

Despite clear guidance from the European Association of Urology (EAU) and the National Institute for Health and Care Excellence (NICE) advising against routine ureteric stenting after uncomplicated ureteroscopy (URS), stents are still widely used [1, 2]. Surveys report that up to 92% of urologists continue to insert stents in these circumstances, indicating a significant discrepancy between guidelines and real‐world practice [3]. Stents are considered necessary in situations involving ureteric injury, obstruction, severe oedema, or sepsis risk. However, their use following an otherwise uncomplicated URS remains a matter of debate. Defining ‘uncomplicated URS’ is therefore crucial. Hiller et al. [4] proposed consensus criteria: unilateral, retrograde URS in patients with American Society of Anesthesiologists (ASA) Physical Status Classification System score <3, no anatomical abnormality, no active infection, no trauma or perforation, and no need for second‐look procedures.

Despite this, practice variation persists. A Cochrane review found evidence on stenting vs omission was of very low to low‐moderate certainty, with conflicting results regarding analgesic use, unplanned hospital visits, and quality of life [5]. Some studies suggest omitting stents reduces emergency visits and improves recovery. Yet, Bhatt et al. [3] showed that most urologists still use stents in the majority of uncomplicated cases, highlighting a gap between evidence and behaviour. We systematically review surgeons’ motivations for stenting after uncomplicated URS and examine patient experiences of living with and removing stents.

The review protocol was registered prospectively on the International Prospective Register of Systematic Reviews (PROSPERO: CRD42023456075). The ‘Enhancing transparency in reporting the synthesis of qualitative research’ (ENTREQ) and Preferred Reporting Items for Systematic Reviews and Meta‐Analyses (PRISMA) guidelines were followed [6, 7]. A structured PubMed/MEDLINE search was performed to August 2023, supplemented by hand‐searching reference lists. Titles and abstracts were screened independently by three reviewers, with disagreements resolved by consensus. Data were extracted and synthesised narratively. Risk of bias in qualitative studies was assessed using the Critical Appraisal Skills Programme (CASP) checklist. Included studies examined surgeon motivations for stenting after uncomplicated URS using qualitative or survey methods or reported patient experiences with indwelling ureteric stents. Eligible designs included randomised trials, surveys, interviews, and observational studies. Reviews, non‐human studies, children aged <12 years, and purely quantitative papers on stenting rates without explanatory data were excluded.

In our results, four studies met the inclusion criteria: one international surgeon survey and three qualitative studies examining patient experiences. Through which two themes emerged.

**Theme 1: surgeons’ motivations for stenting outlined in one study:** Bhatt et al. [3] conducted a global survey of 468 urologists, with 303 full responses, on post‐URS drainage practices. Although guidelines discourage routine stenting, 67% of respondents placed stents in at least half of uncomplicated cases, and a subset always used stents in these cases. Key motivations included: concern for ureteric oedema (77%), fear of residual fragments (43%), case complexity (90%) and procedure duration (46%), and past experience with complications and adherence to traditional teaching (21–24%). Interestingly, >70% reported they would prefer a stent for themselves or a family member after URS, reflecting risk aversion and prioritisation of perceived safety. Despite acknowledging weak evidence, 81% expressed willingness to join a randomised controlled trial (RCT) comparing outcomes in stented vs non‐stented patients.

**Theme 2: patient experiences with stents outlined by three studies:** these are the three studies from the STudy to Enhance uNderstanding of sTent‐associated Symptoms (STENTS) cohort evaluated patient perspectives [8, 9, 10]. Symptom trajectory: Harper et al. [8] documented daily postoperative diaries from 40 patients with stents after URS. Pain intensity and urinary bother were highest in the early days, although pain persisted longer than urinary symptoms. Variability was substantial: some described tolerable discomfort, while others reported severe, disabling pain. Functional and psychological impact: Corneli et al. [9] and Dombeck et al. [10] used interviews to describe the broad impact of stents. Moderate cases reported disturbed mood, difficulty at work, and lifestyle restrictions, while the substantial group described insomnia, depression, inability to perform daily activities, and withdrawal from social/sexual activities. Removal anxiety was common, with some patients describing it as worse than stone colic, while others reported reassurance when supported by staff.

Together, these studies reveal the significant psychological and functional burden of stents, contrasting with surgeons’ preference for routine use.

This review highlights a striking disconnect: surgeons frequently stent uncomplicated URS to mitigate perceived risks, while patients bear the symptomatic and psychological burden. Current EAU and NICE guidelines recommend against routine stenting in uncomplicated cases [1, 2]. Yet, surveys reveal that most urologists continue the practice, often out of concern for rare complications rather than evidence‐based necessity. This suggests that surgeon decision‐making is guided by risk aversion and anecdotal experience, rather than published data.

The patient burden is considerable, with up to 80% of patients reporting reduced quality of life with indwelling stents, including pain, urinary frequency, haematuria, and sexual dysfunction. Psychological consequences include anxiety, depression, and reduced social participation. Removal adds another layer of distress [9]. From health economic impact, urolithiasis management in the UK costs up to £324 million annually, with routine stenting adding further expense. A cost‐analysis confirmed that selective omission is more economical than routine stenting [11].

The persistence of stenting despite evidence and guidelines reflects broader challenges in adhering to clinical practice guidelines. Surgeons’ willingness to participate in trials indicates recognition that high‐quality evidence could drive change. At the same time, better communication with patients about risks, symptoms, and removal could improve experiences and potentially reduce unnecessary stenting.

Regarding limitations, our review included only four eligible studies, which limited its generalisability. Patient experiences were all drawn from one study cohort. Nevertheless, the synthesis reveals consistent themes: surgeons prioritise safety, while patients face disproportionate morbidity.

In conclusion, routine stenting after uncomplicated URS persists despite guideline recommendations against it. Concerns for oedema and obstruction drive surgeon decision making, while patients experience significant pain, urinary bother, psychological distress, and impaired quality of life. Bridging this gap requires more substantial evidence from RCTs, enhanced patient education, and updated guidelines with more explicit definitions. Aligning surgical decision‐making with patient‐centred outcomes could reduce unnecessary morbidity and healthcare costs.

## Disclosure of Interests

None declared.

## Ethics Statement

No ethical approval was required as no patient and/or public were involved in the development of the research aims, methodology and the distribution of this review.

## Author Contributions

Nikita Bhatt and Kevin Gerard Byrnes conceptualised the study. Protocol drafted by Israa Hussein, search strategy developed and drafted by Lyn Mair and Israa Hussein. Israa Hussein, Alexander BCD Ng and Ranil Johann Boaz conducted the abstract and full text review. Israa Hussein, Alexander BCD Ng and Ranil Johann Boaz contributed to data extraction and synthesis. Israa Hussein and Simona Ippoliti contributed to writing and editing the final manuscript. With thanks to Lyn Mair, Clinical Liaison Librarian, for assistance in conducting the literature searches and Medical Subject Headings (MeSH) terms.

## References

[1] Türk C , Petřík A , Sarica K et al. EAU guidelines on urolithiasis. Eur Urol 2016; 69: 475–482 26344917 10.1016/j.eururo.2015.07.041

[2] NICE . Renal and ureteric stones: assessment and management. BJU Int 2019; 123: 220–232 30656839 10.1111/bju.14654

[3] Bhatt NR , MacKenzie K , Shah TT et al. Survey on ureteric drainage after uncomplicated URS. BJUI Compass 2020; 2: 115–125 35474887 10.1002/bco2.48 PMC8988693

[4] Hiller SC , Daignault‐Newton S , Rakic I et al. Appropriateness criteria for ureteral stent omission. Urol Pract 2022; 9: 253–263 36051638 10.1097/upj.0000000000000302 PMC9432824

[5] Ordonez M , Hwang EC , Borofsky M et al. Ureteral stent versus no stent after ureteroscopy. Cochrane Database Syst Rev 2019; 2019: CD012703 10.1002/14651858.CD012703.pub2 PMC6365118 30726554

[6] Tong A , Flemming K , McInnes E et al. ENTREQ: reporting synthesis of qualitative research. BMC Med Res Methodol 2012; 12: 181 23185978 10.1186/1471-2288-12-181 PMC3552766

[7] Page MJ , McKenzie JE , Bossuyt PM et al. PRISMA 2020 statement. BMJ 2021; 372: n71 33782057 10.1136/bmj.n71 PMC8005924

[8] Harper JD , Desai AC , Antonelli JA et al. Quality of life impact after stent insertion: stents daily surveys. BMC Urol 2022; 22: 53 35387623 10.1186/s12894-022-01004-9 PMC8988384

[9] Corneli A , Dombeck C , McKenna K et al. Patient voice and stent removal experiences from stents study. J Endourol 2023; 37: 642–653 37021358 10.1089/end.2022.0810 PMC10280172

[10] Dombeck C , Scales CD , McKenna K et al. Patients' experiences with the removal of a ureteral stent: insights from in‐depth interviews with participants in the USDRN STENTS qualitative cohort study. Urology 2023; 178: 26–36. 37149059 10.1016/j.urology.2023.04.018 PMC10530092

[11] Seklehner S , Sievert KD , Lee R , Engelhardt PF , Riedl C , Kunit T . Cost analysis of stenting in uncomplicated URS. Int Urol Nephrol 2017; 49: 753–761 28197765 10.1007/s11255-017-1538-6

